# Bridging Epilepsy and Cognitive Impairment: Insights from EEG and Clinical Observations in a Retrospective Case Series

**DOI:** 10.3390/jpm15090413

**Published:** 2025-09-02

**Authors:** Athanasios-Christos Kalyvas, Nikoletta Smyrni, Panagiotis Ioannidis, Nikolaos Grigoriadis, Theodora Afrantou

**Affiliations:** 2nd Department of Neurology, AHEPA University Hospital, Aristotle University of Thessaloniki, Stilponos Kyriakidi Street 1, 54636 Thessaloniki, Greece; kalthaxr@hotmail.com (A.-C.K.); nicolsmy7@gmail.com (N.S.); ispanagi@auth.gr (P.I.); ngrigoriadis@auth.gr (N.G.)

**Keywords:** Alzheimer’s disease, late-onset epilepsy, temporal epilepsy, cognitive impairment, dementia, mild cognitive impairment, electroencephalography, epileptiform discharges

## Abstract

**Background**: Epilepsy and cognitive impairment frequently coexist, yet their relationship remains complex and insufficiently understood. This study aims to explore the clinical and electrophysiological features of patients presenting with both conditions in order to identify patterns that may inform more accurate diagnosis and effective management within a personalized medicine framework. **Methods**: We retrospectively analyzed 14 patients with late-onset epilepsy and coexisting cognitive impairment, including mild cognitive impairment and Alzheimer’s disease. Clinical history, cognitive assessments, neuroimaging, and electroencephalographic recordings were reviewed. EEG abnormalities, seizure types, and treatment responses were systematically documented. **Results**: Patients were categorized into two groups: (1) those with established Alzheimer’s disease who later developed epilepsy and (2) those in whom epilepsy preceded cognitive impairment. Temporal lobe involvement was a key feature, with EEG abnormalities frequently localizing to the frontal–temporal electrodes and manifesting as background slowing, focal multiform slow waves, and epileptiform discharges. Levetiracetam was the most commonly used antiseizure medication, and it was effective across both groups. **Conclusions**: This case series highlights the value of EEG in characterizing patients with subclinical and overt epileptic activity and cognitive impairment comorbidity. The inclusion of a substantial number of cases with documented EEG abnormalities provides valuable insight into the interplay between epilepsy and neurodegenerative diseases. By integrating neurophysiological data with clinical and cognitive trajectories, this work aligns with the principles of precision medicine, facilitating a more comprehensive evaluation and tailored management approach. Further longitudinal studies are required to validate prognostic markers and guide optimal therapeutic strategies.

## 1. Introduction

Dementia and epilepsy are significant public health concerns, particularly among elderly populations. The prevalence of dementia increases with age, ranging from an estimated 6% in individuals over 60 years of age to 9.6% in those over 70 years old. Furthermore, the global number of individuals living with dementia is projected to nearly triple by 2050 [1,2,3]. Among the various causes of dementia, Alzheimer’s disease (AD) is the most prevalent, accounting for 60–70% of cases, followed by vascular dementia, Lewy body disease, Parkinson’s disease with dementia, frontotemporal dementia (FTD), and other less common etiologies [4,5]. Mild cognitive impairment (MCI), a potential precursor to dementia, affects approximately 15.56% of individuals aged 50 years or older [6]. Given that up to 39% of these individuals may progress to overt dementia, MCI has garnered significant interest as a critical window for early intervention to mitigate dementia risk [6,7].

Epilepsy, with a prevalence of 1.5% among individuals aged 75 years or older, occurs twice as frequently in elderly populations compared to younger adults [8]. Late onset epilepsy of unknown etiology (LOEU) has emerged as a topic of particular interest due to its association with many neurodegenerative diseases leading to dementia [9]. In the context of AD and LOEU, this association is supported by shared risk factors and pathogenetic mechanisms, leading to the recognition of an epileptic subtype of AD where epilepsy may serve as an early clinical manifestation [10]. This relationship has stimulated substantial research efforts aimed at identifying biomarkers and developing early intervention strategies to prevent irreversible structural and functional changes in the central nervous system [10]. Apart from increasing the overall risk for dementia and the risk for AD, LOEU has been linked to an elevated risk for vascular dementia [11].

The occurrence of epilepsy in individuals with dementia or prior to dementia onset necessitates careful evaluation in clinical practice. In this case series, we present data from 14 patients with comorbid AD or MCI and epilepsy, predominantly LOEU. These cases offer insights into the phenotypic and electroencephalographic characteristics of such patients and underscore the importance of comprehensive diagnostic evaluation and longitudinal monitoring. We cannot overstate the importance of recognizing the comorbidity of late-onset epilepsy and dementia as a distinct clinical entity, necessitating individualized assessment and management to achieve optimal patient outcomes. By tailoring diagnostic and therapeutic approaches to the unique profile of each patient, clinicians may optimize outcomes and improve the quality of life for this vulnerable population.

## 2. Materials and Methods

This study is a retrospective, descriptive case series conducted at the 2nd Neurology Department of AHEPA University General Hospital of Thessaloniki, reviewing clinical and electrophysiological data of patients evaluated between 2019 and 2024. We included patients who were admitted to our clinic following emergency department and outpatient department visits and were diagnosed with both epilepsy and cognitive impairment.

### 2.1. Patient Selection

The patient sample was selected retrospectively from a pool of over 1000 individuals assessed in our department over the past five years. Inclusion criteria were: (1) diagnosis of late-onset epilepsy (including epileptic myoclonus) based on clinical presentation and/or electroencephalography (EEG) findings; (2) concurrent or subsequent diagnosis of cognitive impairment, including MCI or AD; and (3) availability of complete EEG, cognitive assessment, and imaging records. Patients were excluded if clinical seizures were not confirmed or if insufficient data were available.

### 2.2. Ethics Approval and Consent

This study was conducted in accordance with the Declaration of Helsinki. Ethical review and approval were waived for this retrospective case series based on local regulations, as the analysis relied on anonymized clinical data. Nevertheless, all patients (or their legal representatives) provided written informed consent for the use of their clinical data for research purposes.

### 2.3. Clinical and Cognitive Assessment

Each patient underwent a standardized clinical neurological examination and cognitive evaluation using the Greek-adapted version of the Addenbrooke’s Cognitive Examination-Revised (ACE-R) [12]. Scores were used to assess the degree of cognitive impairment.

### 2.4. Electroencephalography

All patients underwent EEG evaluation using the international 10–20 system. Hyperventilation was performed for three minutes, and intermittent photic stimulation was applied at 5, 10, 15, and 20 Hz. Surface electrodes were placed peri-orbitally to record eye movements. Electrocardiographic activity was also captured.

### 2.5. Neuroimaging

Brain magnetic resonance imaging (MRI) or computerized tomography (CT) scans were performed in all patients to assess structural abnormalities. Single-photon emission computed tomography (SPECT) was conducted using technetium-99m-labeled hexamethylpropyleneamine oxime (Tc-99m HMPAO) to evaluate regional cerebral blood flow (rCBF), aiding in dementia subtype diagnosis.

### 2.6. Cerebrospinal Fluid Analysis

When clinically indicated, cerebrospinal fluid (CSF) analysis was performed to assess biomarkers for Alzheimer’s disease pathology, including amyloid-β, total tau, and phosphorylated tau (p-tau). Additional CSF studies were conducted when necessary to exclude alternative diagnoses.

### 2.7. Data Analysis

Patients were divided into two groups:

• Group 1: Patients with established diagnosis of AD who later developed epilepsy (n = 6).
• Group 2: Patients who presented initially with epilepsy and were subsequently diagnosed with AD or MCI (n = 8).

Demographic data, seizure types, EEG findings, ACE-R scores, and treatment responses were collected and analyzed descriptively. Due to the limited sample size, no inferential statistical testing was performed. The results are presented as means or frequencies.

## 3. Results

This case series comprised 14 patients with comorbid cognitive decline and epilepsy. Table 1 provides demographic data and follow-up durations. The mean age at presentation was 66.9 years (range: 52–88), and the mean follow-up period was 42.1 months. The study population included 6 males and 8 females.

Two primary patient groups were identified. Group 1 (n = 6) included individuals with a prior diagnosis of AD who subsequently developed epilepsy (Table 2). Group 2 (n = 8) consisted of patients in whom epileptic seizures were the initial clinical manifestation, later followed by a diagnosis of AD or MCI (Table 3). Group 1 had a slightly higher mean age (67.5 years) and lower mean ACE-R scores (35.5), suggesting more advanced cognitive impairment at the time of seizure onset compared to Group 2 (mean age 66.0 years; mean ACE-R score 56.3). In Group 1, female patients predominated (4 out of 6), whereas in Group 2, the sex distribution was equal.

Seizure semiology also differed between groups. Myoclonus was the predominant seizure type in Group 1 (4 of 6 cases), while focal impaired awareness seizures of temporal origin were more common in Group 2 (7 of 8 cases). All patients achieved seizure control with one or two antiseizure medications, and none progressed to drug-resistant epilepsy.

EEG abnormalities were present in all patients, with patterns localizing primarily to the temporal or frontotemporal regions. Common findings included generalized background slowing, frontal intermittent rhythmic delta activity (FIRDA), polymorphic slow waves (2–5 Hz), atypical complexes of spike-wave, and sharply contoured epileptiform discharges. Table 2 and Table 3 summarize the seizure semiology, EEG findings, and treatment modalities for each case. A comparative overview of demographic, clinical, cognitive, and EEG characteristics between the two groups is provided in Table 4, highlighting key distinctions in seizure type, cognitive status, and electrophysiological profiles.

### 3.1. Epileptic Seizures Following Established AD

This section presents six patients who developed epileptic seizures after receiving a confirmed diagnosis of AD. These cases illustrate the emergence of various seizure types—predominantly myoclonus and focal seizures—in the context of progressive neurodegeneration, as well as EEG characteristics and treatment responses.

#### 3.1.1. Patient 1

**Figure 1:**
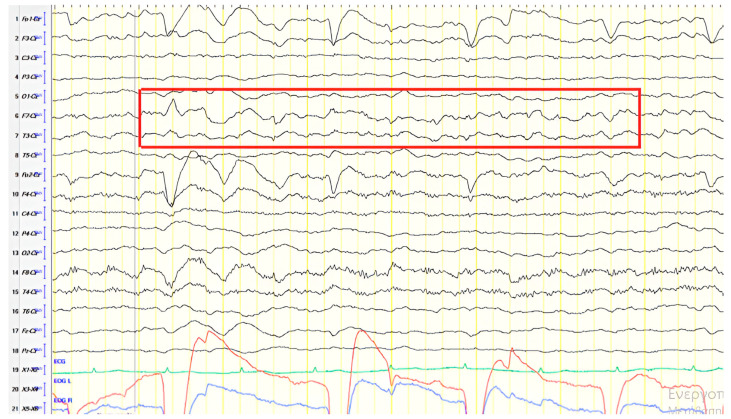
Cz referential montage showing generalized background slowing (6 Hz) with slow activities 4–5 Hz over the frontal–temporal regions, with predominance in the left hemisphere (red frame).

A 57-year-old female with a three-year history of cognitive decline presented with recent-onset myoclonic jerks in the upper extremities. Neurological examination revealed brisk deep tendon reflexes (DTRs), a positive grasp reflex, apraxia in the upper extremities, alexia, agraphia, and visuospatial impairment. EEG demonstrated generalized background slowing (8 Hz) without focal abnormalities. The ACE-R score was 38, rCBF SPECT supported an AD diagnosis, and rivastigmine was initiated. Three years later, worsening cognitive decline and generalized myoclonus prompted repeat EEG, which revealed further generalized background slowing (6 Hz) and slow-wave activity (4–5 Hz) over the frontal–temporal regions, more pronounced on the left (Figure 1). Levetiracetam was introduced, with myoclonus improvement, and a gastrostomy tube was inserted to manage feeding difficulties.

#### 3.1.2. Patient 2

**Figure 2:**
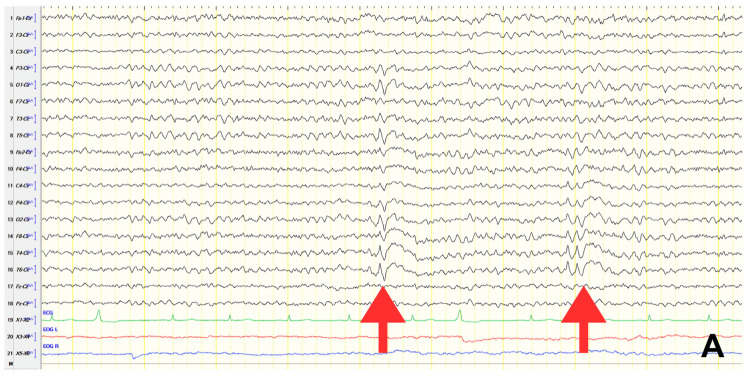

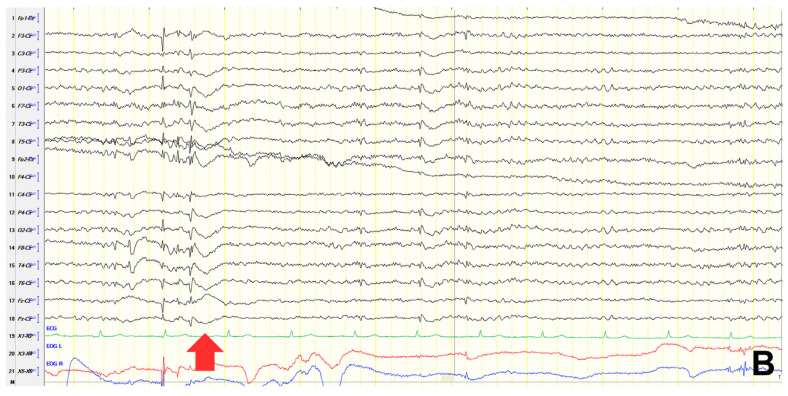

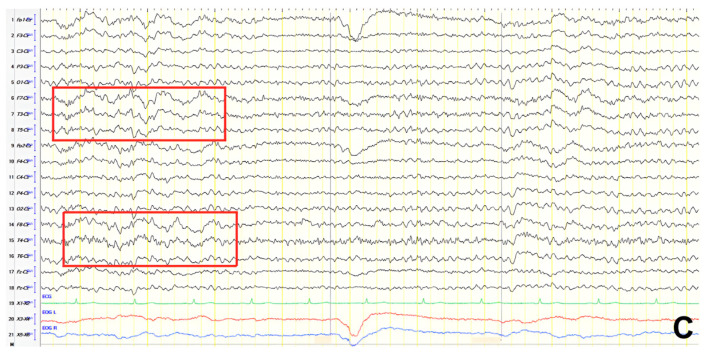
Cz referential montage showing (**A**) slow wave activity with rare complexes of slow spike and wave 2–2.5 Hz over the posterior regions predominating in the right hemisphere (red arrows), (**B**) mild generalized background slowing with improvement of paroxysmal activities; muscle artifact during myoclonus was followed by a slow wave (red arrow), (**C**) slow polymorphic activity 2.5–4 Hz over the temporal–posterior areas (red frames).

A 70-year-old male with a 2.5-year history of cognitive and behavioral impairment presented with myoclonic jerks affecting all extremities and the face. Neurological examination demonstrated brisk DTRs and positive primitive reflexes. ACE-R score was 44. Brain MRI revealed parietal atrophy, while EEG showed slow wave activity and rare complexes of slow spike-wave discharges (2–2.5 Hz) predominantly in the right posterior regions (Figure 2A). Myoclonic jerks corresponded to muscle artifacts (followed by slow waves in the EEG) without identifiable spike anomalies on EEG (Figure 2B). Levetiracetam was added to donepezil with subsequent EEG improvement (Figure 2C) and resolution of myoclonus. rCBF SPECT findings were consistent with logopenic-variant AD.

#### 3.1.3. Patient 3

**Figure 3:**
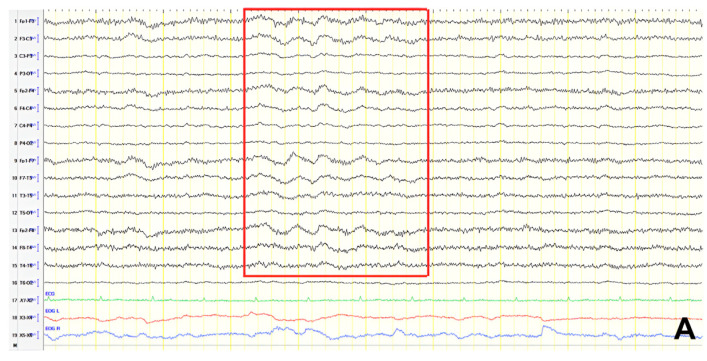

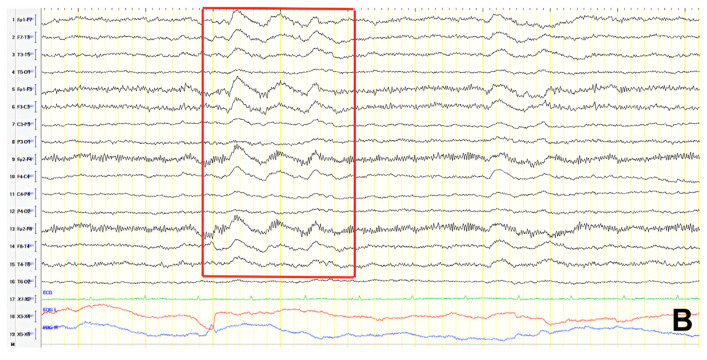
Double banana bipolar montage showing (**A**) generalized background slowing (6–7 Hz) with continuous polymorphic slow discharges 2.5–3 Hz over the frontal–temporal regions, recognized as frontal intermittent rhythmic delta activity (red frame), (**B**) generalized background slowing (6–7 Hz) with continuous polymorphic slow discharges 2.5–3 Hz over the frontal–temporal areas, predominating in the left hemisphere (red frame).

A 61-year-old female presented with a six-year history of progressive memory loss and behavioral changes. Examination revealed temporal disorientation, visuospatial deficits, apraxia, brisk DTRs, and positive primitive reflexes. ACE-R score was 10. Brain CT showed significant posterior atrophy, and rCBF SPECT confirmed AD. Initial EEG recorded generalized background slowing (6–7 Hz) and continuous polymorphic slow discharges (2.5–3 Hz) over the frontal–temporal regions, recognized as frontal intermittent rhythmic delta activity (FIRDA) (Figure 3A). The patient was diagnosed with AD, and donepezil was initiated. Distal upper extremity myoclonus developed 16 months later. Repeat EEG findings were consistent with previous results, with greater left-hemispheric involvement (Figure 3B). Clonazepam was initiated, resulting in marked improvement.

#### 3.1.4. Patient 4

**Figure 4:**
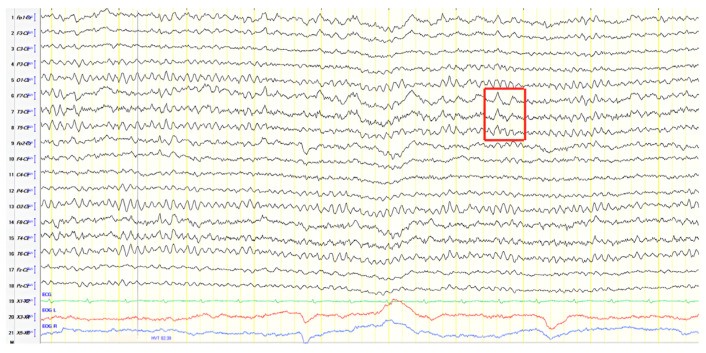
Cz referential montage showing occasional continuous paroxysmal polymorphic slow-sharp activity 3–4 Hz over the left frontal–temporal region (red frame).

A 62-year-old male with a one-year history of mild memory issues and episodic speech disturbance underwent evaluation. Neurological examination was notable for positive bilateral palmomental reflexes. ACE-R score was 69. Brain MRI revealed mild cortical atrophy, and rCBF SPECT supported an AD diagnosis. EEG recorded occasional continuous paroxysmal slow-sharp waves (3–4 Hz) over the left frontal–temporal region (Figure 4). Oxcarbazepine was prescribed, as the clinical course suggested a possible causal role of subclinical temporal epileptic activity in the observed episodic speech disturbance.

#### 3.1.5. Patient 5

**Figure 5:**
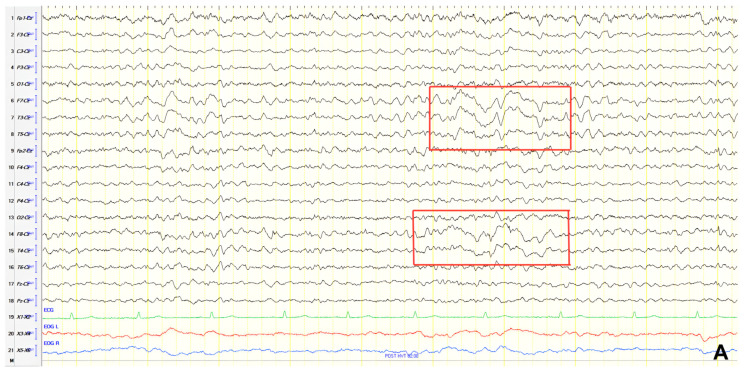

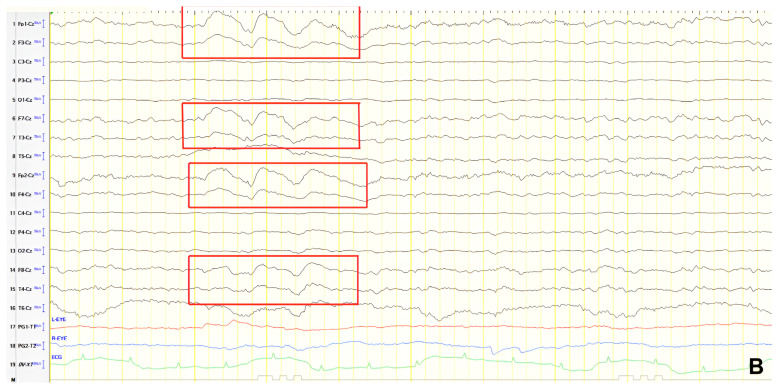
Cz referential montage showing (**A**) mild generalized background slowing 7–8 Hz with frequent polymorphic slow activity 3–5 Hz over the frontal–parietal regions, predominating in the left hemisphere, where they appear in a sharply contoured morphology during hyperventilation (red frames), (**B**) generalized background slowing (7 Hz) and frontal intermittent rhythmic delta activity (red frames).

A 67-year-old female presented with speech and memory complaints. Neurological examination revealed brisk DTRs, positive primitive reflexes, and anomic aphasia. ACE-R score was 66. Brain MRI showed mild cortical atrophy, and rCBF SPECT confirmed AD. EEG demonstrated mild generalized background slowing (7–8 Hz) with frequent sharply contoured slow polymorphic activity (3–5 Hz) predominantly over the left frontal–parietal regions (Figure 5A). She was diagnosed with logopenic-variant AD, and rivastigmine was initiated. Myoclonus leading to frequent falls developed four years later. Cognition was deteriorated at that time (ACE-R score 32), and EEG revealed generalized slowing (7 Hz), FIRDA predominantly in the left hemisphere, and isolated slow-sharp waves over the anterior frontal areas. Levetiracetam was initiated and improved myoclonus. Two years later, the patient developed non-convulsive status epilepticus requiring hospitalization and treatment adjustment. EEG showed generalized slowing (7 Hz) and FIRDA (Figure 5B). The levetiracetam dose was increased, and a gastrostomy tube was placed.

#### 3.1.6. Patient 6

**Figure 6:**
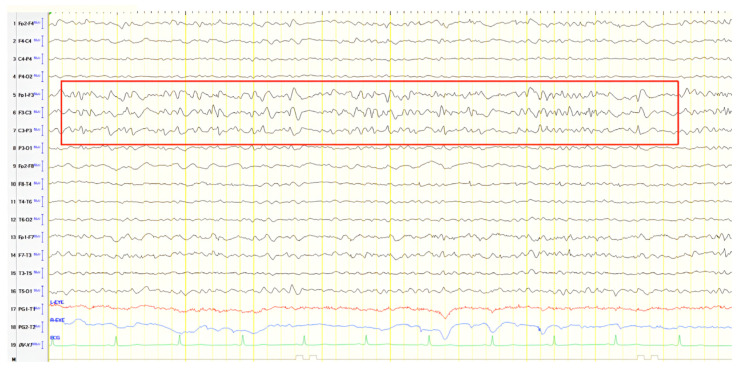
Double banana bipolar montage showing generalized background slowing (6–7 Hz) with paroxysmal biphasic slow-sharp epileptiform activity 2.5–3 Hz over the left frontal–central region (red frame).

An 88-year-old female with long-standing AD presented following three episodes of unresponsiveness, head turning, mouth automatisms, and staring. Two more similar episodes occurred in the emergency department. Two years earlier, she had a generalized seizure attributed to hyperglycemia, leading to a diagnosis of diabetes and initiation of levetiracetam. Neurological examination revealed temporal and spatial disorientation. ACE-R score was 20. EEG recorded generalized background slowing (6–7 Hz) with paroxysmal biphasic slow-sharp epileptiform discharges (2.5–3 Hz) over the left frontal–central region (Figure 6). During hospitalization, the levetiracetam dose was increased, and lamotrigine was added. The patient remained seizure-free at the one-year follow-up.

Table 2 summarizes the seizure types, EEG findings, and antiepileptic therapy in Group 1.

### 3.2. Epileptic Seizures Preceding or Coinciding with AD/MCI Diagnosis

In this section, we describe eight patients for whom epileptic activity represented an early finding, preceding a later diagnosis of AD or MCI or recognized at the same time with it. These cases highlight the potential of epilepsy to serve as an early indicator of neurodegenerative processes and underscore the clinical importance of longitudinal cognitive assessment in patients with late-onset epilepsy.

#### 3.2.1. Patient 7

**Figure 7:**
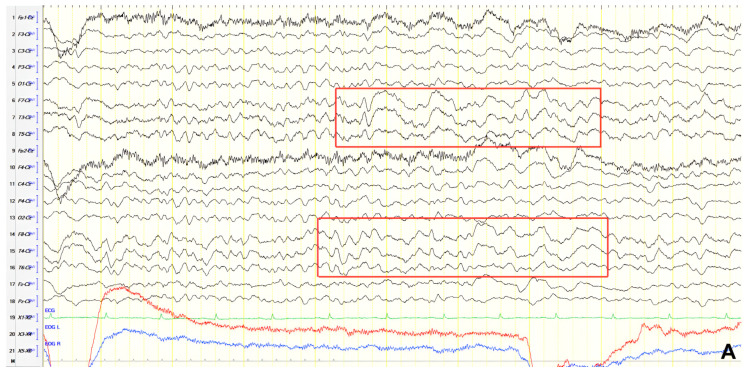

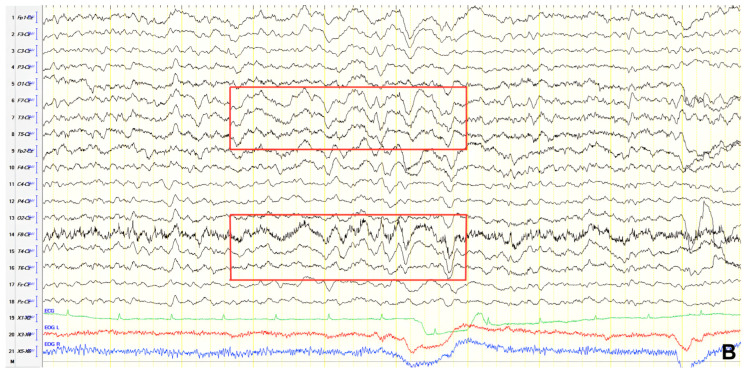
Cz referential montage showing (**A**) generalized background slowing (7–8 Hz), with continuous polymorphic slow waves 2–3 Hz over the frontal–temporal regions, occasionally presenting as atypical complex of spike-wave at 2 Hz in the left hemisphere (red frames), (**B**) generalized background slowing (7–8 Hz) and paroxysmal polymorphic slowing with atypical complex of spike-wave 2–2.5 Hz over the left frontal–temporal area (red frames).

A 65-year-old male with a known epilepsy diagnosis, treated with valproate, presented following a fall. He exhibited mental slowing, logopenic speech, brisk DTRs, and bilateral positive grasp reflexes. ACE-R score was 40. Neuroimaging, including brain CT and MRI, demonstrated diffuse atrophy, and rCBF SPECT findings were consistent with advanced AD. Initial EEG recorded generalized background slowing (7–8 Hz), with continuous polymorphic slow waves (2–3 Hz) over the frontal–temporal regions, occasionally presenting as atypical complexes of spike-wave discharges at 2 Hz in the left hemisphere (Figure 7A). Valproic acid dosage was adjusted, as the clinical course suggested the occurrence of focal impaired awareness seizure of temporal origin, resulting in a follow-up EEG showing slight improvement (Figure 7B). The patient was diagnosed with AD, with cognitive impairment onset following temporal lobe epilepsy development.

#### 3.2.2. Patient 8

**Figure 8:**
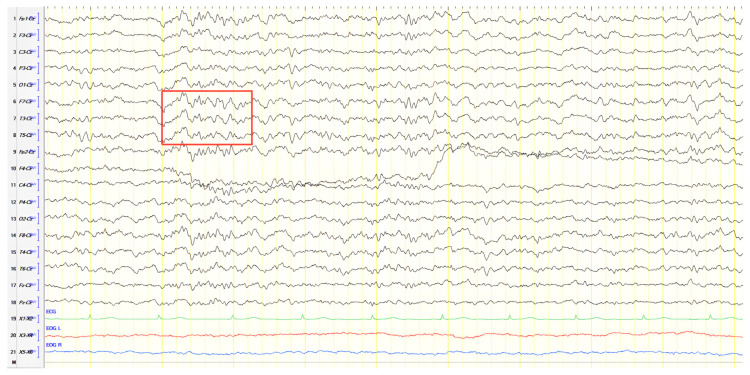
Cz referential montage showing slow-sharp waves 6 Hz over the left anterior temporal region (red frame).

A 72-year-old female presented to the emergency department with status epilepticus, successfully managed with intravenous diazepam and phenytoin. According to relatives, the patient had been unresponsive with staring spells, followed by convulsions starting in the right face and arm, progressing to generalized convulsions, and culminating in bilateral convulsive status epilepticus. Postictal findings included somnolence, disorientation, and Todd’s paresis affecting the right upper and lower limbs. These characteristics were compatible with focal onset evolving into bilateral convulsive status epilepticus. History revealed two epileptic episodes during sleep in the previous 1.5 years: a generalized tonic–clonic seizure and status epilepticus. Treatment with levetiracetam and phenytoin was then initiated but later discontinued. Relatives also reported recent memory impairment and disorientation. Neurological examination showed brisk DTRs and positive grasp reflexes. ACE-R score was 56. Brain CT revealed global atrophy, and EEG demonstrated slow-sharp waves at 6 Hz over the left anterior temporal region (Figure 8). A diagnosis of MCI in the context of epilepsy was made, and levetiracetam dosage was increased. Follow-up EEGs at 6 and 18 months revealed no epileptiform abnormalities.

#### 3.2.3. Patient 9

**Figure 9:**
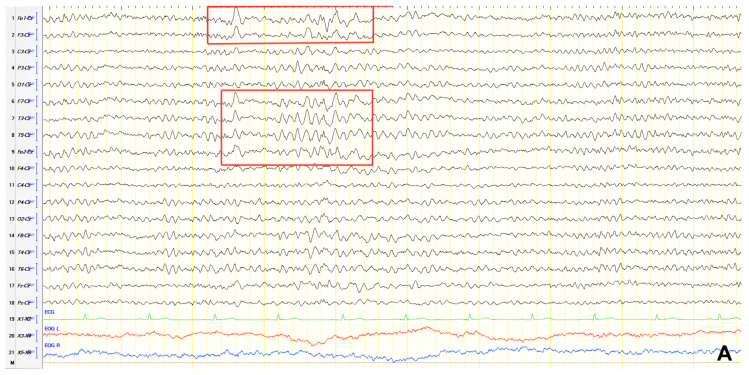

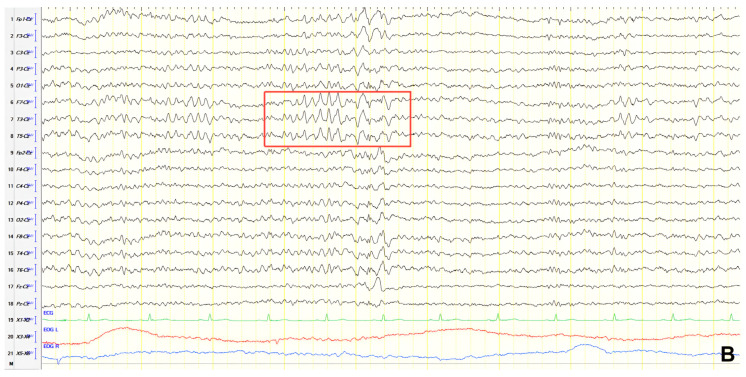

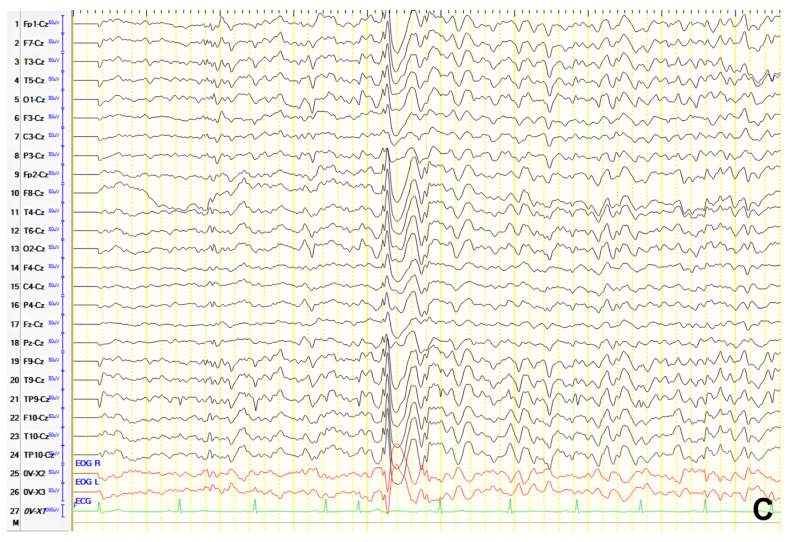
(**A**) Cz referential montage showing transient multiform slow sharply contoured activity over the left frontal–temporal region (4–5 Hz) (red frames), (**B**) Cz referential montage showing rare transient slow sharply contoured polymorphic activity 4–5 Hz over the left temporal and frontal–temporal areas (red frame), (**C**) Cz referential montage showing focal epileptiform discharge over the left frontal–temporal area rapidly progressing into a brief generalized burst.

A 52-year-old male with chronic epilepsy presumed secondary to prior encephalitis presented with attention lapses. Initial EEG (20 years earlier) showed slow, sharply contoured discharges over the left frontal–temporal region, MRI demonstrated bilateral thalamic signal abnormalities without contrast enhancement, and he had been on valproate ever since (Figure 9A). Follow-up EEG conducted 10 years later recorded similar activity over the left temporal and frontal–temporal areas (Figure 9B). MRI and EEG normalized over time. Upon presentation, neurological examination revealed mild mental slowing and brisk DTRs, and ACE-R score was 78. A 24 h video EEG revealed left frontal–temporal epileptiform discharges evolving to brief generalized bursts (Figure 9C). The patient was diagnosed with focal impaired awareness seizures of temporal origin and MCI. Perampanel was added to valproate. Over a six-year follow-up, the patient remained seizure-free and clinically stable.

#### 3.2.4. Patient 10

**Figure 10:**
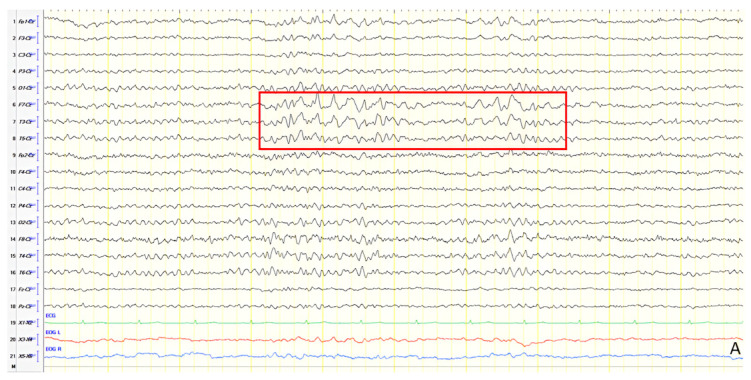

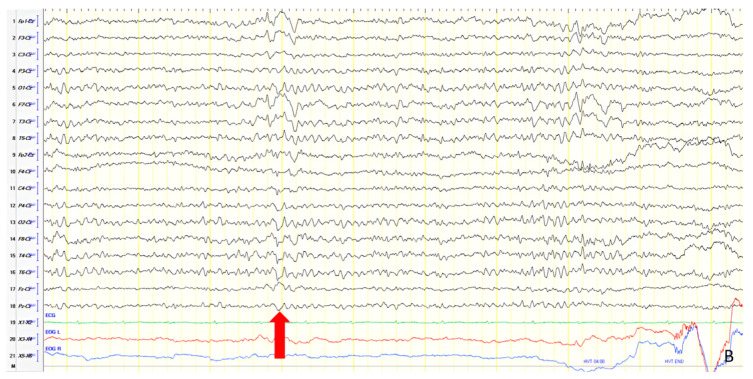

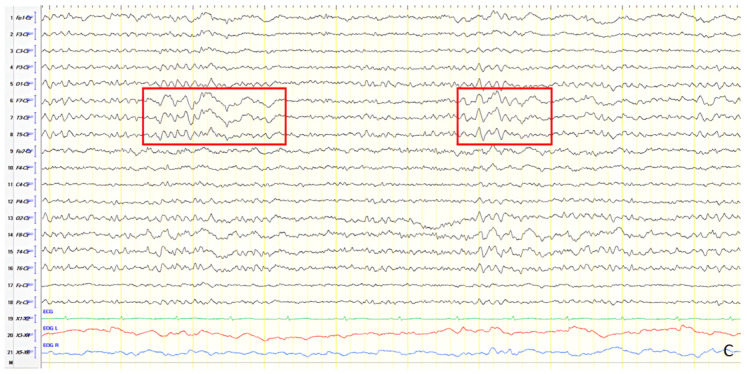

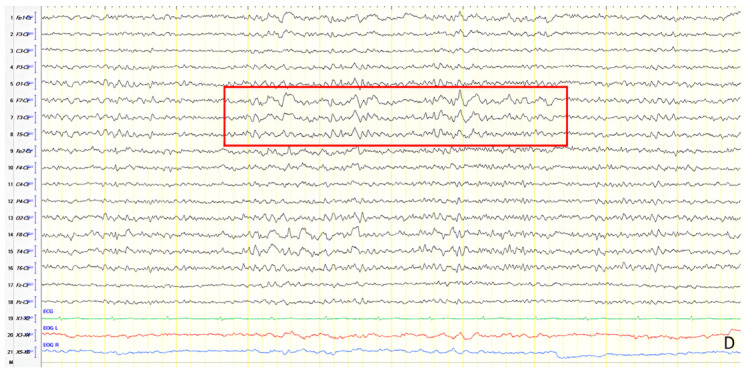
Cz referential montage showing (**A**) frequent paroxysmal slow polymorphic activity 4–6 Hz over the left frontal and anterior temporal regions, occasionally exhibiting a sharply contoured morphology (red frame), (**B**) paroxysmal slow polymorphic activity 1.5–2 Hz over the left anterior temporal area (red arrow) (6-month follow-up), (**C**) slow polymorphic activity 4–5 Hz with duration of 0.5–1 s over the left anterior temporal area (red frames) (13-month follow-up), (**D**) slow polymorphic activity 4–5 Hz accompanied by slow sharp waves over the left anterior temporal area (red frame) (18-month follow-up).

A 64-year-old female presented with memory impairment, sleep disturbances, and transient episodes with mild confusion. She had a history of an epileptic seizure causing a fall with loss of consciousness 18 months earlier, followed by episodes of unresponsiveness. Initial EEG revealed frequent paroxysmal slow polymorphic activity (4–6 Hz) over the left frontal and anterior temporal regions, occasionally exhibiting a sharply contoured morphology (Figure 10A). Levetiracetam was initiated with clinical improvement. Neurological examination was normal except for brisk DTRs, and ACE-R score was 78, leading to a diagnosis of MCI. Brain MRI was unremarkable, but EEG findings remained consistent with previous recordings. Levetiracetam was gradually replaced with oxcarbazepine after the patient reported aggressiveness, but due to poor tolerance, lacosamide was introduced as an adjunct therapy to a low dose of levetiracetam. Follow-up EEGs over 18 months revealed evolving patterns of paroxysmal slow polymorphic activity (Figure 10B–D). The patient’s cognition deteriorated over time, and CSF biomarkers confirmed AD pathology.

#### 3.2.5. Patient 11

**Figure 11:**
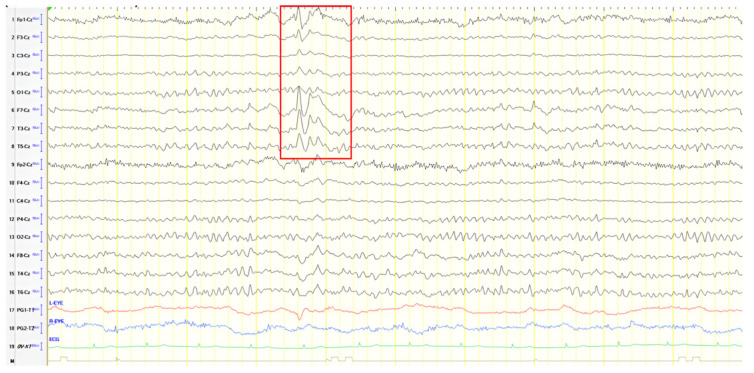
Cz referential montage showing paroxysmal multiform slow-spiky activity 2–3 Hz over the anterior temporal areas with left predominance (red frame).

A 74-year-old female presented with an episode of altered consciousness lasting eight minutes, during which she was unresponsive and staring. History revealed similar shorter episodes and memory complaints. Neurological examination identified positive grasp and palmomental reflexes. ACE-R score was 70, indicating MCI. Brain MRI revealed significant frontal and temporal atrophy. EEG recorded paroxysmal multiform slow-spiky activity (2–3 Hz) predominating in the left anterior temporal areas (Figure 11). A diagnosis of focal impaired awareness seizures of left temporal origin and MCI was made, and levetiracetam was initiated.

#### 3.2.6. Patient 12

**Figure 12:**
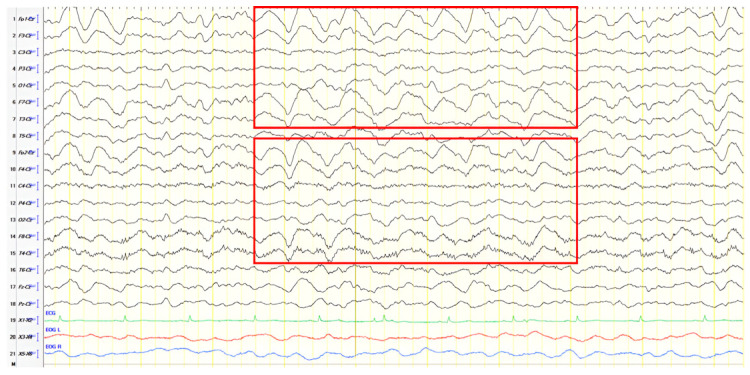
Cz referential montage showing generalized background slowing (7 Hz) with continuous recording of slow polymorphic activity, rarely of a triphasic morphology, 2–3 Hz over the frontal–temporal areas with predominance in the left hemisphere and evolving into frontal intermittent rhythmic delta activity bilaterally during hyperventilation (red frames).

A 63-year-old female presented with three years of memory, speech, and behavioral impairment and a history of prior unmedicated epileptic seizures. Neurological examination revealed apraxia, and ACE-R score was 18. Brain MRI and rCBF SPECT findings were consistent with AD. EEG revealed generalized background slowing (7 Hz) with continuous recording of slow polymorphic activity, rarely of a triphasic morphology (2–3 Hz) over the frontal–temporal areas, predominantly in the left hemisphere and evolving into FIRDA bilaterally during hyperventilation (Figure 12). The patient was diagnosed with AD in the context of pre-existing epilepsy, but she was not offered antiepileptic treatment in the absence of clinical seizures and epileptiform activity on EEG.

#### 3.2.7. Patient 13

**Figure 13:**
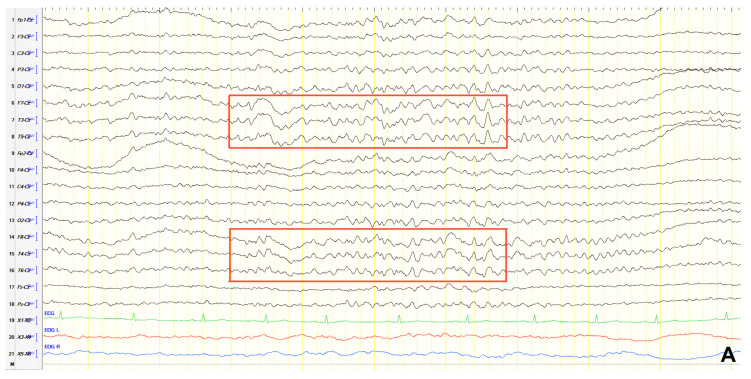

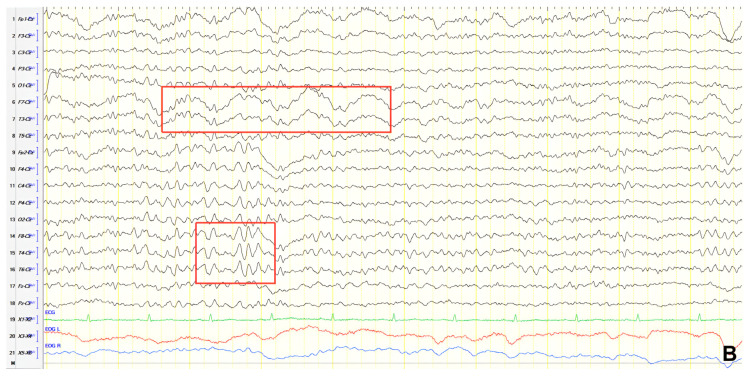

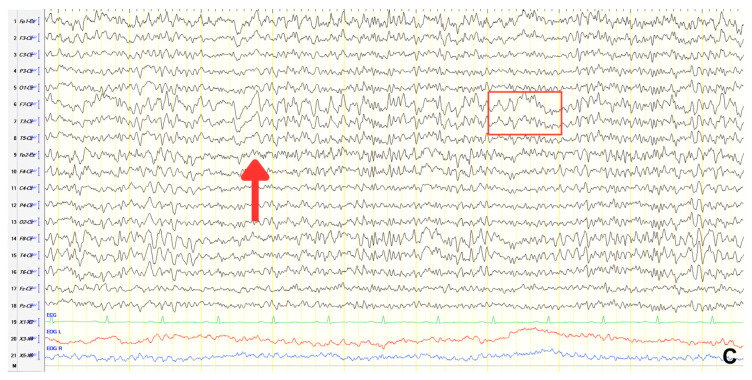

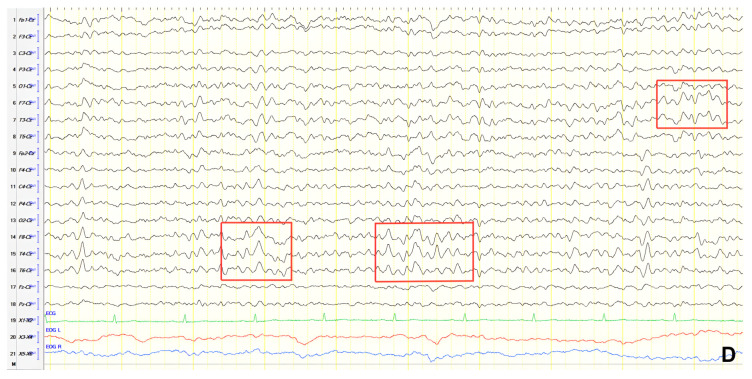
Cz referential montage showing (**A**) generalized background slowing with slow waves 3–5 Hz, particularly over the frontal–temporal areas (red frames), (**B**) continuous multiform sharply contoured slow waves 2–4 Hz over the left frontal–temporal area along with transient slow multiform abnormality 4 Hz over the right temporal area (red frames), (**C**) isolated sharp waves over the left frontal–temporal area (red frame) with occasional focal slow waves 2–3 Hz in the left hemisphere (red arrow), (**D**) frequent paroxysmal multiform slow abnormalities 2–3.5 Hz over the frontal–temporal areas (red frames).

**Figure 14:**
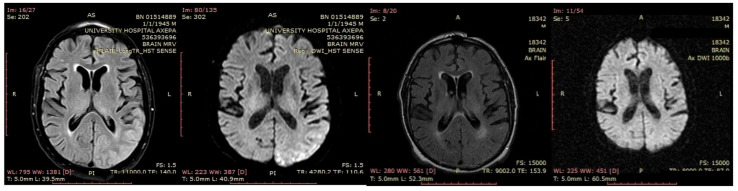
From left to right: the first and the second images demonstrated signal abnormality in the cortex of the left temporal–parietal–occipital areas in fluid-attenuated inversion recovery (FLAIR) and diffusion-weighted imaging (DWI), respectively, which resolved in follow-up MRI (third image—FLAIR, fourth image—DWI).

A 70-year-old male was initially diagnosed with MCI. At that time, ACE-R score was 83, and the EEG showed generalized slowing with slow waves (3–5 Hz) predominantly in the frontal–temporal areas (Figure 13A). Two years later, he experienced multiple episodes of unresponsiveness and staring, followed by confusion, along with episodes characterized by fluctuating speech impairment characterized as aphasia, coinciding with febrile illness. EEG recorded continuous multiform sharply contoured slow waves 2–4 Hz over the left frontal–temporal area with transient slow multiform abnormality 4 Hz over the right temporal area (Figure 13B). Brain MRI revealed abnormal signal in the cortex of the left temporal–parietal–occipital areas, attributed to ischemic changes secondary to prolonged epileptic discharges (Figure 14). He was treated with levetiracetam and lacosamide, with clinical improvement, and the subsequent EEG 3 days later revealed isolated sharp waves over the left frontal–temporal area with occasional focal slow waves 2–3 Hz in the left hemisphere (Figure 13C). Six months later, he was reevaluated. Neurological examination demonstrated apraxia, apathy, paraphasia, and positive grasp and palmomental reflexes. Cognitive testing yielded an ACE-R score of 71. MRI abnormality was resolved (Figure 14) and repeat EEG was invariable (Figure 13D). The clinical course, including the resolution of MRI abnormalities and the stabilization of cognitive decline following seizure control, suggested that the epileptic activity was causally related to cognitive impairment in this patient from early on. Consequently, he was diagnosed with focal impaired awareness seizures of left temporal origin, leading to MCI.

#### 3.2.8. Patient 14

**Figure 15:**
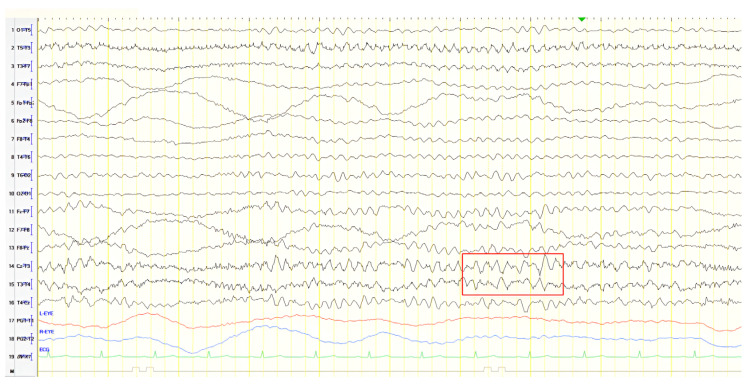
Double banana combined with transverse bipolar montage showing paroxysmal sharply contoured, slow polymorphic activity 2.5–3 Hz over the left frontal–temporal region (red frame).

A 68-year-old male with a history of levetiracetam treatment after an epileptic seizure 3 months earlier presented after a second episode of transient loss of consciousness lasting 10 min, without overt convulsions or other motor phenomena. Initial EEG recorded frequent transient theta waves in the posterior temporal areas, evolving into subclinical rhythmic electrographic discharge of adults (SREDA) during drowsiness. Neurological examination upon presentation detected positive primitive reflexes, and ACE-R score was 41. No reliable cognitive history was available, owing to the absence of relatives. Brain CT and MRI were remarkable for mild atrophy. Repeat EEG showed paroxysmal sharply contoured, slow polymorphic activity (2.5–3 Hz) over the left frontal–temporal region (Figure 15). Levetiracetam dosage was increased, and rCBF SPECT established the diagnosis of AD in the context of temporal epilepsy.

In Table 3, a summary of patients 7–14, detailing seizure types, EEG findings, and treatment, is provided.

## 4. Discussion

Epilepsy and dementia are prevalent in elderly populations and necessitate timely and accurate diagnosis and treatment. Given the increasing emphasis on personalized approaches to neurological disorders, understanding the bidirectional relationship between epilepsy and cognitive impairment is essential for developing tailored diagnostic and therapeutic strategies. Contrary to earlier perceptions, epilepsy is no longer viewed solely as a complication of late-stage dementia [13]. Particularly in AD, an epileptic phenotype has been proposed, where LOEU serves as an initial or prominent symptom during the early stages, often correlating with poorer patient outcomes [14]. Shared risk factors and common pathogenetic mechanisms underpin the association between AD and epilepsy, suggesting that disruptions in excitatory–inhibitory balance contribute to cognitive dysfunction and seizure development [10,15]. In this retrospective case series, we analyzed 14 patients with comorbid epilepsy and cognitive impairment to explore the clinical and electrophysiological profiles that characterize this intersection. Our findings contribute to precision medicine efforts by highlighting clinical and electroencephalographic biomarkers that may aid in the early detection of neurodegenerative diseases in patients with late-onset epilepsy, and vice versa.

In our case series, in Group 1, myoclonic seizures were the most common seizure type, occurring in 4 of 6 patients. Other seizure types included episodic speech disturbances, non-convulsive status epilepticus, and focal impaired awareness seizures of temporal origin. Focal seizures with impaired awareness are the most common seizure type in AD [16]. Myoclonus, particularly in late-stage AD, can occur in up to half of patients [17]. The predominance of myoclonus in our AD patients may reflect a more advanced stage of AD at the time of seizure onset in our patients, as suggested by lower ACE-R scores (mean: 29.8 in myoclonus cases vs. 44.5 in others) or the subclinical nature of many focal seizures with impaired awareness, which may go unnoticed [18]. However, we acknowledge that myoclonus can also result from non-epileptic causes, including metabolic disturbances. In our cases, the epileptic origin was supported by cortical EEG abnormalities, clinical improvement with antiseizure medication, and the absence of metabolic derangements on extensive diagnostic evaluation. Nonetheless, confirmation with advanced neurophysiological techniques such as polygraphic EEG and back-averaging would have strengthened these observations.

In Group 2, all patients with a clearly defined seizure type exhibited focal impaired awareness seizures originating from the temporal lobe. One notable case (Patient 8) progressed to bilateral convulsive status epilepticus, underscoring the potential severity of undiagnosed or untreated focal seizures in this population. Focal impaired awareness seizures without prominent motor signs—such as episodes of unresponsiveness, behavioral arrest, or speech disturbances—were observed in the majority of cases (Patients 8, 10, 11, 13, and 14). These subtle manifestations are frequently under-recognized in older adults and may be misattributed to psychiatric or neurodegenerative conditions, leading to diagnostic delays [19]. The predominance of this seizure type in our cohort is consistent with prior studies describing the semiological profile of LOEU, where seizures often originate in the temporal lobe and present without convulsions [20,21]. This reinforces the importance of clinical vigilance and systematic EEG evaluation in elderly patients with cognitive symptoms, particularly in the absence of overt motor phenomena. Accurate identification of epileptic etiology is critical, as seizure control can lead to stabilization or even improvement in cognitive function, further underscoring the therapeutic significance of timely diagnosis [22].

The majority of patients (9/13) achieved seizure control with monotherapy, while four required dual therapy, consistent with literature reporting effective seizure control in up to 90% of cases with a single agent [20,21,23]. This is particularly significant given the advanced age and comorbidities of these patients, which can limit treatment options [10]. In Group 1, patients were treated with antiseizure medications including levetiracetam, clonazepam, oxcarbazepine, or lamotrigine, while in Group 2, the antiseizure medications used included levetiracetam, valproate, perampanel, lacosamide, and oxcarbazepine. Levetiracetam was the most frequently used antiseizure medication (9/14), favored for its favorable tolerability and safety. Notably, emerging studies have explored levetiracetam’s potential neuroprotective effects in early-stage AD, suggesting its dual role in seizure suppression and cognitive stabilization [14,21,24,25,26].

At the time of first seizure evaluation, background EEG rhythms were slightly slower in Group 1 (mean 7.4 Hz) compared to Group 2 (mean 8.6 Hz). Although not statistically significant, this trend is consistent with literature linking background rhythm slowing to the severity of cognitive decline [27,28,29]. In Group 1, EEG abnormalities included FIRDA, polymorphic delta activity, paroxysmal slow-sharp epileptiform activity, and slow spike-wave discharges. In Group 2, EEG findings consistently showed temporal epileptiform activity, while other EEG findings included polymorphic delta activity, slow-sharp waves, and FIRDA. The predominance of temporal involvement aligns with previous studies on LOEU and its proposed association with prodromal Alzheimer’s pathology [30]. EEG abnormalities implicating temporal regions were present in 13 of the 14 patients analyzed, including patterns that emphasize the value of EEG in detecting subclinical epileptiform activity that may contribute to fluctuating cognitive symptoms, thereby overlapping with cognitive decline due to AD and complicating treatment decisions [31,32].

MCI represents a state of cognitive dysfunction that does not significantly impair daily activities but carries a high risk of progression to dementia. Among patients with LOEU, 59% exhibit MCI, further increasing the likelihood of dementia compared to cognitively intact LOEU patients [10,21]. Decreased cerebrospinal fluid (CSF) amyloid-β levels in LOEU patients with MCI suggest an underlying AD pathology, reinforcing the concept of an “epileptic prodromal AD”, where epilepsy precedes dementia by more than 2.5 years [23,33,34]. Detection of the presence of cognitive impairment is crucial in patients with epilepsy, as recent studies confirm a beneficial effect of seizure control in cognitive outcomes [10]. In our department, cognitive evaluation in patients with LOEU is routinely implemented, with extended diagnostic workup applied in cognitively affected individuals.

To better characterize the temporal relationship between epilepsy and cognitive impairment, we analyzed the time interval between the onset of seizures and the clinical diagnosis of MCI or AD in both groups. On average, epileptic seizures preceded the diagnosis of MCI or AD by 6.7 years in Group 2. In Group 2, 3 out of 8 patients (patients 7, 12, 14) were diagnosed with AD, with the first seizure occurring 5 years, 25 years, and 3 months prior to dementia onset (mean 10.1 years) respectively. In MCI patients 8, 9, and 10, the first seizure occurred 18 months, 20 years, 19 months earlier, respectively, while in patients 11 and 13, MCI was diagnosed at the time of the evaluation for the first epileptic episode (mean 4.6 years). The small number of patients prevents us from drawing robust conclusions. However, this variation emphasizes the heterogeneity of disease progression and supports existing literature suggesting that epilepsy may precede or accelerate cognitive decline in certain individuals. Vossel et al. reported seizures preceding the diagnosis in 83% of patients with either amnestic MCI or AD, while epilepsy diagnosis was established earlier or around the time of amnestic MCI or AD diagnosis in 51% of patients [35]. In contrast, in Group 1, where AD preceded epilepsy, the interval ranged from 1 to 7.3 years (mean: 4 years). This comparison highlights the variability in disease trajectories, emphasizing that epilepsy may either emerge as a late manifestation in established dementia or serve as an early harbinger of future cognitive decline.

Patient 9, with longstanding epilepsy potentially linked to encephalitis, illustrates the complexity of attributing cognitive impairment to either epileptic or neurodegenerative causes. In such cases, chronic epilepsy itself or post-infectious changes may be responsible for cognitive decline, independent of AD pathology [36,37,38,39].

Case 13 illustrates the diagnostic complexity of distinguishing epilepsy-related cognitive impairment from early neurodegenerative decline. Initially classified as having MCI, the patient demonstrated fluctuating cognitive and speech symptoms in the setting of epileptiform activity localized to the left temporal lobe. Over time, repeated EEGs revealed persistent focal epileptiform discharges, while MRI abnormalities emerged in the corresponding cortical areas. However, these changes proved reversible: after the introduction of antiseizure medications, both the MRI abnormalities and the patient’s cognitive performance stabilized, with no further deterioration noted over six months of follow-up. This clinical course strongly suggests that epilepsy—rather than an underlying neurodegenerative process—was the primary driver of cognitive symptoms. The reversibility of imaging and clinical findings aligns with the concept that seizure-related metabolic dysfunction, rather than neuronal loss, can underlie apparent cognitive decline [40]. Notably, this echoes the work of Lam et al., who demonstrated that hippocampal hyperactivity in AD patients may be present from the early stages and that this dysfunction could be ameliorated by levetiracetam [41]. Therefore, this case highlights the importance of considering epilepsy as a modifiable contributor to cognitive dysfunction in older adults and underscores the diagnostic and therapeutic value of serial EEG and neuroimaging in such presentations. In line with Lam et al.’s and Vossel et al.’s findings, it raises the possibility that a subset of patients diagnosed with MCI or even early AD may, in fact, have epilepsy-related cognitive symptoms amenable to treatment [41,42,43,44]. Recognizing this distinction is essential for delivering targeted therapy and avoiding premature labeling of irreversible neurodegeneration.

Despite these insights, our study has certain limitations. Several patients were evaluated prior to the COVID-19 pandemic and were lost to follow-up, limiting our understanding of long-term outcomes. The observational nature and small sample size restrict causal inferences. While seizure control appeared to stabilize cognition in select patients, this finding requires validation in prospective studies investigating whether early therapeutic intervention in late-onset epilepsy can modify the trajectory of cognitive decline.

Nevertheless, this case series contributes meaningful clinical observations on the intersection of epilepsy and cognitive impairment. A notable strength is the integration of clinical, neuroimaging, and electrophysiological data—including EEG images—enabling a nuanced characterization of this comorbidity. These cases help systematize the interplay between epilepsy and cognitive decline, shedding light on how seizure activity may either contribute to or coexist with neurodegeneration. By documenting a spectrum of clinical presentations, EEG abnormalities, and disease trajectories, our findings highlight the importance of comprehensive diagnostic evaluation of epilepsy in older adults, particularly LOEU. Additionally, we highlight the need for meticulous follow up in those presenting with cognitive symptoms in order to facilitate timely identification of epileptic activity. Furthermore, our data emphasize the importance of tailored antiepileptic treatment strategies, as demonstrated by the varying responses to different pharmacological interventions across patients.

In conclusion, our data suggest that epileptic activity may precede or exacerbate cognitive impairment in older adults. Early recognition and appropriate treatment of seizures may offer an opportunity to mitigate cognitive decline in this population. Multimodal assessment, including EEG, imaging, and cognitive testing, enhances precision and provides a framework for clinicians toward more individualized management of patients with comorbid epilepsy and cognitive impairment. Future longitudinal studies are essential to better delineate causal pathways and assess whether early therapeutic intervention can alter the clinical trajectory of these patients.

## Figures and Tables

**Table 1:** Demographic and cognitive characteristics of patients.

Patient	Age (Years)	Gender	Follow Up (Months)	ACE-R Score
1	57	F	33	38
2	70	M	7	44
3	61	F	16	10
4	62	M	1.5	69
5	67	F	60	32
6	88	F	10	20
7	65	M	0.3	40
8	72	F	18	56
9	52	M	228	78
10	64	F	84	76
11	74	F	6	70
12	63	F	-	18
13	70	M	41	71
14	68	M	-	41

M: male, F: female, ACE-R: Addenbrooke’s Cognitive Examination—Revised.

**Table 2:** Summary of epileptic seizure types, electroencephalographic findings, and anti-epileptic treatment in patients 1–6.

Patient	Seizure Type	EEG Findings	Medication
1	Myoclonus	– Generalized slowing, slow activities 4–5 Hz over the frontal–temporal areas, predominance in the left hemisphere	Levetiracetam
2	Myoclonus	– Slow wave activity with rare atypical complexes of slow spike and wave 2–2.5 Hz over the posterior regions, predominance in the right hemisphere. – Mild generalized slowing, muscle artifact during myoclonus followed by a slow wave – Slow polymorphic activity 2.5–4 Hz over the temporal–posterior areas	Levetiracetam
3	Myoclonus	– Generalized slowing with continuous polymorphic slow discharges 2.5–3 Hz over the frontal–temporal areas, recognized as FIRDA – Generalized slowing with continuous slow polymorphic discharges 2.5–3 Hz over the frontal–temporal areas, predominance in the left hemisphere.	Clonazepam
4	Focal without impaired awareness of temporal origin (episodic speech problems)	– Occasional continuous paroxysmal slow-sharp epileptiform activity 3–4 Hz over the left frontal–temporal area.	Oxcarbazepine
5	(1) Myoclonus (2) Non-convulsive status epilepticus	– Mild generalized slowing with frequent polymorphic slow activity 3–5 Hz over the frontal–parietal areas, predominance in the left hemisphere, where they appear in a sharply contoured morphology during hyperventilation. – Generalized slowing and FIRDA.	Levetiracetam
6	Focal impaired awareness seizure of temporal origin	– Generalized slowing with paroxysmal biphasic slow-sharp epileptiform activity 2.5–3 Hz over the left frontal–central area.	Levetiracetam, Lamotrigine

EEG: electroencephalogram, Hz: Hertz, FIRDA: frontal intermittent rhythmic delta activity.

**Table 3:** Summary of epileptic seizure types, electroencephalographic findings, and anti-epileptic treatment in patients 7–14.

Patient	Seizure Type	EEG Findings	Medication
7	Focal impaired awareness seizure of temporal origin	– Generalized slowing, with continuous polymorphic slow waves 2–3 Hz over the frontal–temporal regions, occasionally presenting as atypical complex of spike-wave at 2 Hz in the left hemisphere – Generalized background slowing and paroxysmal polymorphic slowing with atypical complex of spike-wave 2–2.5 Hz over the left frontal–temporal area.	Valproate
8	Focal onset evolving into bilateral convulsive status epilepticus	– Slow-sharp waves 6 Hz over the left anterior temporal region.	Levetiracetam
9	Focal impaired awareness seizure of temporal origin	– Transient multiform slow sharply contoured activity over the left frontal–temporal region (4–5 Hz). – Rare transient slow sharply contoured polymorphic activity 4–5 Hz over the left temporal and frontal–temporal areas. – Focal epileptiform discharge over the left frontal–temporal area rapidly progressing into a brief generalized discharge.	Valproate, Perampanel
10	Focal impaired awareness seizure of temporal origin	– Frequent paroxysmal slow polymorphic activity 4–6 Hz over the left frontal and anterior temporal regions, occasionally exhibiting a sharply contoured morphology. – Paroxysmal slow polymorphic activity 1.5–2 Hz over the left anterior temporal area. – Slow polymorphic activity 4–5 Hz with a duration of 0.5–1 s over the left anterior temporal area. – Slow polymorphic activity 4–5 Hz accompanied by slow sharp waves over the left anterior temporal area.	Lacosamide, Levetiracetam
11	Focal impaired awareness seizure of left temporal origin	– Paroxysmal multiform slow-spiky activity 2–3 Hz over the anterior temporal areas with left predominance.	Levetiracetam
12	Unclassified	– Generalized background slowing with continuous recording of slow polymorphic activity, rarely of a triphasic morphology, 2–3 Hz over the frontal–temporal areas with predominance in the left hemisphere and evolving into frontal intermittent rhythmic delta activity bilaterally during hyperventilation.	-
13	Focal impaired awareness seizure of left temporal origin	– Generalized background slowing with slow waves 3–5 Hz, particularly over the frontal–temporal areas. – Continuous multiform sharply contoured slow waves 2–4 Hz over the left frontal–temporal area along with transient slow multiform abnormality 4 Hz over the right temporal area. – Isolated sharp waves over the left frontal–temporal area with occasional focal slow waves 2–3 Hz in the left hemisphere. – Frequent paroxysmal multiform slow abnormalities 2–3.5 Hz over the frontal–temporal areas.	Levetiracetam, Lacosamide
14	Focal impaired awareness seizure of temporal origin	– Paroxysmal sharply contoured, slow polymorphic activity 2.5–3 Hz over the left frontal–temporal region.	Levetiracetam

EEG: electroencephalogram, Hz: Hertz,

**Table 4:** Comparison between Group 1 and Group 2.

Characteristic	Group 1: AD Before Epilepsy (n = 6)	Group 2: Epilepsy Before AD/MCI (n = 8)
Mean age (years)	67.5	66
Sex (males/females)	2/4	4/4
Mean ACE-R score	35.5	56.3
Most common seizure type	Myoclonus (4/6)	Temporal focal impaired awareness (7/8)
EEG—Most frequent pattern	• Polymorphic delta • Slow-sharp waves • Slow-spike and wave • FIRDA	• Focal epileptiform in temporal areas • Polymorphic delta • Slow sharp waves
Background rhythm mean frequency	7.4 Hz	8.6 Hz
Patients with temporal lobe EEG focus	6/6	7/8
Common ASMs used	Levetiracetam	Levetiracetam, Valproate
Response to monotherapy	5/6	4/7 (one patient excluded)
Mean interval between diagnoses	4 years (range: 1–7.3)	6.7 years overall (10.1 years (AD)/4.6 years (MCI))

AD: Alzheimer Disease, MCI: Mild Cognitive Impairment, ACE-R: Addenbrooke’s Cognitive Examination—Revised, EEG: Electroencephalography, Hz: Hertz, FIRDA: Frontal Intermittent Rhythmic Delta Activity, ASMs: antiseizure medications.

## Data Availability

The original contributions presented in this study are included in the article. Further inquiries can be directed to the corresponding author.

## Abbreviations

ACE-R	Addenbrooke’s Cognitive Examination-Revised
AD	Alzheimer’s Disease
BiPLEDS	Bilateral Independent Periodic Lateralized Epileptiform Discharges
CSF	Cerebrospinal Fluid
CT	Computed Tomography
DTRs	Deep Tendon Reflexes
EEG	Electroencephalography
FDG-PET	Fluorodeoxyglucose-Positron Emission Tomography
FIRDA	Frontal Intermittent Rhythmic Delta Activity
MCI	Mild Cognitive Impairment
MRI	Magnetic Resonance Imaging
rCBF SPECT	Regional Cerebral Blood Flow Single-Photon Emission Computed Tomography
SREDA	Subclinical Rhythmic Electrographic Discharge of Adults
